# Comparison of DVH‐based plan verification methods for VMAT: ArcCHECK‐3DVH system and dynalog‐based dose reconstruction

**DOI:** 10.1002/acm2.12123

**Published:** 2017-06-26

**Authors:** Masahide Saito, Noriyuki Kadoya, Kiyokazu Sato, Kengo Ito, Suguru Dobashi, Ken Takeda, Hiroshi Onishi, Keiichi Jingu

**Affiliations:** ^1^ Department of Radiology University of Yamanashi Yamanashi Japan; ^2^ Department of Radiation Oncology Tohoku University School of Medicine Sendai Japan; ^3^ Radiation Technology Tohoku University Hospital Sendai Japan; ^4^ Department of Therapeutic Radiology Tohoku University School of Medicine Sendai Japan

**Keywords:** dose reconstruction, patient QA, radiotherapy, VMAT

## Abstract

The purpose of this study was comparing dose‐volume histogram (DVH)‐based plan verification methods for volumetric modulated arc therapy (VMAT) pretreatment QA. We evaluated two 3D dose reconstruction systems: ArcCHECK‐3DVH system (Sun Nuclear corp.) and Varian dynalog‐based dose reconstruction (DBDR) system, developed in‐house. Fifteen prostate cancer patients (67.6 Gy/26 Fr), four head and neck cancer patient (66 Gy/33 Fr), and four esophagus cancer patients (60 Gy/30 Fr) treated with VMAT were studied. First, ArcCHECK measurement was performed on all plans; simultaneously, the Varian dynalog data sets that contained the actual delivered parameters (leaf positions, gantry angles, and cumulative MUs) were acquired from the Linac control system. Thereafter, the delivered 3D patient dose was reconstructed by 3DVH software (two different calculating modes were used: High Sensitivity (3DVH‐HS) and Normal Sensitivity (3DVH‐NS)) and in‐house DBDR system. We evaluated the differences between the TPS‐calculated dose and the reconstructed dose using 3D gamma passing rates and DVH dose index analysis. The average 3D gamma passing rates (3%/3 mm) between the TPS‐calculated dose and the reconstructed dose were 99.1 ± 0.6%, 99.7 ± 0.3%, and 100.0 ± 0.1% for 3DVH–HS, 3DVH–NS, and DBDR, respectively. For the prostate cases, the average differences between the TPS‐calculated dose and reconstructed dose in the PTV mean dose were 1.52 ± 0.50%, −0.14 ± 0.55%, and −0.03 ± 0.07% for 3DVH–HS, 3DVH–NS, and DBDR, respectively. For the head and neck and esophagus cases, the dose difference to the TPS‐calculated dose caused by an effect of heterogeneity was more apparent under the 3DVH dose reconstruction than the DBDR. Although with some residual dose reconstruction errors, these dose reconstruction methods can be clinically used as effective tools for DVH‐based QA for VMAT delivery.

## 1 INTRODUCTION

Recently, volumetric modulated arc radiotherapy (VMAT) has become a routine technique in many facilities. Although this technique improves the conformity of dose distribution to PTV and reduces the impact on OARs, its use in a complex dose distribution with a sharp gradient necessitates patient‐specific quality assurance (QA). The most frequently employed method for QA has been comparison of the calculated and measured doses in a phantom. Particularly, the point dose on an ion chamber and planar dose distribution on a film are usually measured. In general, gamma analysis has been used to compare measured and calculated dose distributions in a commercial radiation treatment planning system (TPS).^1^ However, these conventional patient‐specific QA procedures are very time consuming for the clinical staff. In addition, some previous studies showed that gamma analysis cannot directly predict the actual patient dose.^2^, ^3^

To tackle these problems, some independent dose reconstruction methods have been proposed to evaluate patient dose–volume histogram (DVH) and dose index for VMAT pretreatment QA. One of the methods is called measurement‐guided dose reconstruction (MGDR), a system which is commercially provided as Matri‐COMPASS (IBA Dosimetry, Schwarzenbruck, Germany), Delta4 anatomy (ScandiDos, Inc., Ashland, VA, USA), and ArcCHECK‐3DVH (Sun Nuclear Corporation, Melbourne, FL, USA). The ArcCHECK‐3DVH system delivers a 3D patient dose that can be reconstructed using 3DVH software from the original TPS plan and ArcCHECK measurement data. The reconstructed dose from this system could be compared with the TPS‐calculated dose using 3D gamma and DVH dose index analyses. The accuracy of the system has already been investigated in several studies.^4^, ^5^, ^6^

Another method is machine log file‐based dose reconstruction. Some have reported the use of log files generated by multi‐leaf collimator (MLC) controller as a tool for DVH‐based dose verification for patient‐specific QA of intensity‐modulated radiotherapy (IMRT).^7^ In VMAT, on the other hand, machine log files that contain other dynamic parameters (gantry angles and cumulative MU) are generated from a Linac control system. Some studies demonstrated dose reconstruction methods that modify TPS plan data using delivered parameters from these log files and recalculate by TPS dose calculation algorithm.^8^, ^9^ Furthermore, Teke et al. showed that VMAT QA based on machine log files could be performed with Monte Carlo simulation.^10^

Therefore, many approaches to DVH‐based QA have been proposed and verified for accuracy. However, there have been few studies that directly compared these different DVH‐based QA systems. Tyagi et al. evaluated the accuracy of dose reconstruction by the ArcCHECK‐3DVH system and the machine log file‐based system on only 1–2 patients for each treatment site.^11^ Therefore, to integrate this DVH‐based patient‐specific QA into clinical practice, we further investigated its accuracy for dose reconstruction. The purpose of this study was to compare two DVH‐based plan verification methods for VMAT pretreatment QA. These two 3D dose reconstruction systems were the ArcCHECK‐3DVH and the Varian dynalog‐based dose reconstruction (DBDR).

## 2 METHODS

### 2.A VMAT plans

Fifteen prostate cancer patients (67.6 Gy/26 Fr), four head and neck cancer patients (66 Gy/33 Fr), and four esophagus cancer patients (60 Gy/30 Fr) treated with VMAT were studied. Single‐ (179°–181°, counter‐clockwise) or double‐ (179°–181°, counter‐clockwise and clockwise) or triple‐ (179°–181°, counter‐clockwise, clockwise, and counter‐clockwise) full arc plans were generated by experienced medical physicists using Eclipse TPS.ver.8.6 (Varian Medical Systems, Palo Alto, CA, USA). The anisotropic analytical algorithm (AAA) ver.11.0.1 with a 2‐mm grid was used for dose calculation. All VMAT plans were delivered by 6 MV or 15 MV X‐ray beams of Varian 23EX with a 120 millennium MLC.

### 2.B ArcCHECK‐3DVH system

The ArcCHECK‐3DVH system (Sun Nuclear Corporation, Melbourne, FL, USA) was commercially available tool for DVH‐based QA. ArcCHECK was cylindrical 3D diode array, which contained 1386 diodes (detector sixe: 0.8 × 0.8 mm^2^) in a helical arrangement at intervals of 10 mm and with diameter of 21 cm. To reconstruct an “actual” 3D patient dose from the measured ArcCHECK data, we used 3DVH software ver. 3.2 that had an internal calculation engine, which was called ArcCHECK planned dose perturbation (ACPDP). To perform ACPDP, the following data set were prepared: reference DICOM RT plan, DICOM RT dose (TPS‐calculated dose for the patient and ArcCHECK geometries, respectively), and ArcCHECK measurement data (.acml). The ACPDP algorithm involved the following calculation steps: (a) synchronizing the planned data with the ArcCHECK virtual inclinometer recorded data; (b) generating a relative 3D dose grid to a homogeneous cylindrical phantom for each sub‐beam; (c) morphing the relative dose based on the ArcCHECK‐measured data to produce the 3D absolute dose in the cylindrical phantom; (d) taking the ratio of the reconstructed dose to the TPS‐calculated dose for each voxel in the phantom; and (e) perturbing the TPS‐calculated dose of the patient by the above ratios. The final grid size of the reconstructed dose was the same as that of the TPS dose calculation. Further details on ACPDP have been described elsewhere.^12^ In addition to ACPDP calculation step 3, two different modes were used in this study; these were High Sensitivity (3DVH–HS) and Normal Sensitivity (3DVH–NS) modes. There were two conditions in which 3DVH–NS dose morphing was dampened per diode: (a) if the dose of the diode was below a qualifying threshold dose and (b) the diode was in a very high gradient region. On the other hand, a high range 3DVH–HS dose morphing was possible even in steep dose gradients for the 4D sub‐beams and in high‐ and low‐dose regions. The 3DVH–HS dose morphing is recommended for detecting even very small deviations from ideal behavior.

### 2.C In‐house dynalog‐based dose reconstruction system

In VMAT, two sets of Varian dynalog were generated. One was beam delivery dynalog, which was created by the Linac console and contained information delivered from the dynamic beam (e.g., the actual cumulative dose delivered (MU) versus the actual gantry angle); these parameters were only recorded for each control point. The other log was the MLC dynalog, which was created by the MLC controller. The file was separately generated and acquired every 50 ms for the MLC banks A and B. Details of the MLC dynalog have been described elsewhere.^13^ In this study, dynalog‐based dose reconstruction (DBDR) was performed by an in‐house software and Eclipse TPS. First, the original DICOM RT plan from Eclipse TPS was modified by the in‐house software using the two dynalog sets. Particularly, control point information of the originally planned parameters (leaf positions, gantry angles, and cumulative MU weights) was rewritten to the actual delivered parameters recorded in the dynalog sets. The in‐house software was developed by Visual C++ and open source DICOM tool kit DCMTK ver. 3.6.0. The DICOM RT plan reconstructed by the software was placed back to Eclipse TPS, and the actual patient 3D dose was recalculated by the TPS dose calculation algorithm (AAA).

### 2.D Validation of the in‐house DBDR system

Before using the in‐house DBDR system for patient‐specific QA, the system was validated by a method similar to the one used by Juan et al. to check for programming errors.^7^ For a baseline plan (Single‐arc prostate VMAT, 2.6 Gy/1 Fr), nine MLC error plans were generated (Table 1). To measure the absolute dose at the center of the phantom, these plans were delivered by a 15‐MV X‐ray beam of Varian 23EX with a 120 millennium MLC to an ArcCHECK phantom with a customized acrylic plug that was holding a 0.6‐cc PTW 30013 Farmer ionization chamber. Absolute isocenter dose was used to evaluate the accuracy of the in‐house DBDR system.

**Table 1:** Comparisons between ion chamber measurement and reconstructed dose by in‐house DBDR system in center of ArcCHECK phantom

	Plan name	D_Meas_		D_DBDR_	
		Gy	Error/Baseline	Gy	Error/Baseline
0	Baseline	2.56	–	2.55	–
1	1 mm MLC Gap opening	2.70	1.05	2.67	1.05
2	1 mm MLC Opening bank A	2.63	1.03	2.59	1.02
3	1 mm MLC Opening bank B	2.65	1.03	2.62	1.03
4	1 mm MLC Gap closing	2.45	0.96	2.44	0.96
5	1 mm MLC Closing bank A	2.52	0.98	2.51	0.98
6	1 mm MLC Closing bank B	2.50	0.98	2.48	0.97
7	1 mm MLC Shift bank A	2.55	1.00	2.52	0.99
8	1 mm MLC Shift bank B	2.58	1.01	2.57	1.01
9	Random error	2.57	1.00	2.55	1.00

D_meas_, absolute dose measured by ion chamber; D_DBDR_, absolute dose calculated by in‐house Dynalog‐based dose reconstruction method.

### 2.E Workflow and Analysis of DVH‐based QA

**Figure 1:**
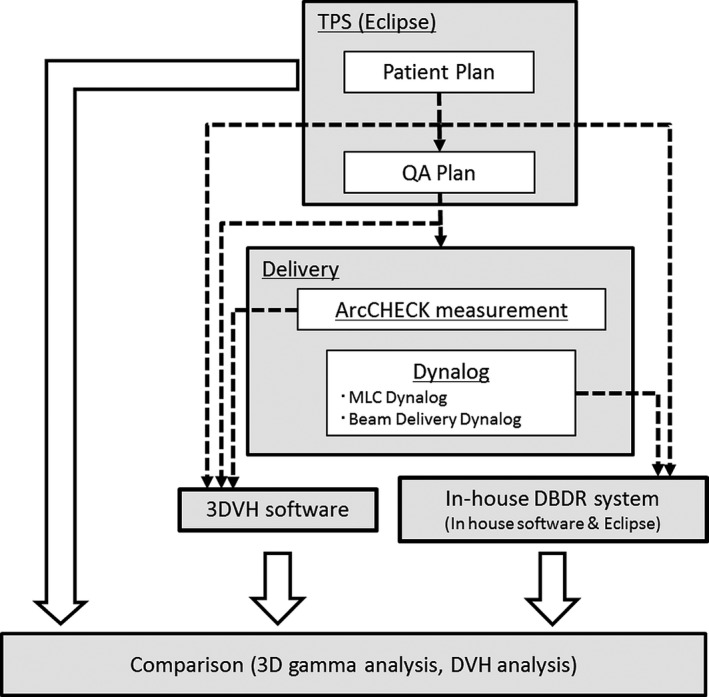
A schematic design of this study. Two 3D patient‐specific QA methods (using ArcCHECK‐3DVH system and in‐house DBDR system) were carried out simultaneously, and the reconstructed doses were compared to TPS‐calculated dose.

A schematic design of this study is shown in Fig. 1. First, ArcCHECK QA plans were created from the original plans for all patients. Second, ArcCHECK measurement (ArcCHECK was calibrated with 200 MU with a 10 × 10 cm^2^ field size at a gantry angle 0° before plan irradiation) was performed on all plans; simultaneously, the Varian dynalog data sets that contained the actual delivered parameters (leaf positions, gantry angles, and cumulative MUs) were acquired from the Linac control system. Thereafter, the delivered 3D patient dose was reconstructed by 3DVH software and in‐house DBDR system. We evaluated the differences between the TPS‐calculated dose and the reconstructed dose using whole body 3D gamma passing rates (3%/3 mm, 2%/2 mm, and 1%/1 mm with global normalization, threshold 10%) and DVH dose index analysis. For the DVH analysis of the prostate case, PTV doses (mean dose, D95% and maximum dose) and rectum wall and bladder wall dose (mean dose, V35 and V55) were evaluated. For the whole neck case, PTV doses (D50% and maximum dose), brainstem and spinal cord doses (maximum dose), and parotids dose (mean dose and maximum dose) were evaluated. For the esophagus case, PTV doses (D50% and maximum dose), spinal cord dose (maximum dose), and lungs doses (mean dose and V20) were evaluated. In addition, we calculated the clinically effective confidence limit values for each DVH dose index using the following eq. (1)

(1) $$\text{Confidence limit}=|{\text{DD}}_{\mathrm{mean}}|+1.96\;{\text{DD}}_{\mathrm{SD}}$$

where DD_mean_ was the average dose differences between the TPS‐calculated dose and the reconstructed dose and DD_SD_ was the standard deviation. All 3D analyses were performed in 3DVH software ver.3.2.

## 3 RESULTS

### 3.A Validation of the in‐house DBDR System

The results of validation of the in‐house DBDR system are summarized in Table 1. Nine MLC error plans (#1~9) were created for baseline plan (#0), and irradiated to 0.6‐cc Farmer ion chamber in center of ArcCHECK phantom. The values for the error/baseline ratio showed a significant positive correlation (R^2^ = 0.986; *P* < 0.01) between the measurement values and DBDR values. Although we validated the system using MLC error plans without accounting for gantry angle and MU errors, we confirmed that the system could work correctly in dynamic irradiation.

### 3.B Analysis of DVH‐based QA

Before 3D reconstructed dose analysis, we evaluated the 2D planar dose that was measured by ArcCHECK for all patients using SNC Patient software ver. 6.6 (Sun Nuclear Corporation, Melbourne, FL, USA). 2D gamma analysis showed good agreement between the measured and calculated planar doses (gamma passing rate >97.5% for all patients (3%/3 mm, global normalization, threshold 10%)), indicating that conventional 2D patient‐specific QA was mostly successful.

**Figure 2:**
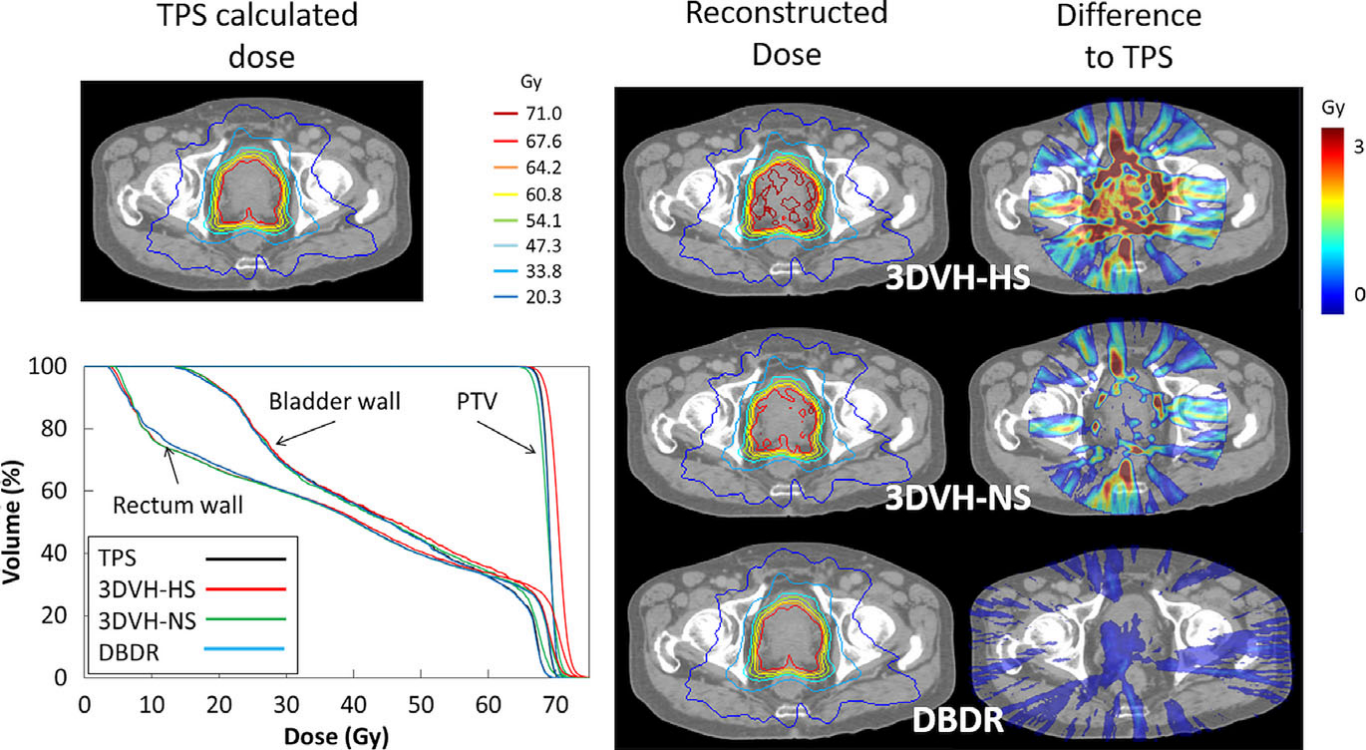
A representative 3D dose validation result (patient 4). Reference TPS‐calculated dose (upper left), DVHs of PTV, rectum wall and bladder wall for each reconstructed dose (bottom left), and each reconstructed dose distribution and the difference maps to TPS‐calculated dose of each method (right).

Figure 2 shows a representative DVH‐based QA for patient 4; the reconstructed dose distribution of each method, the difference map to reference TPS‐calculated dose, and DVHs of this patient are shown. For 3DVH methods, there were differences observed between the TPS‐calculated and reconstructed doses. In addition, a difference between 3DVH–HS and 3DVH–NS was observed. On the other hand, using the in‐house DBDR method, there were little differences between the TPS‐calculated and reconstructed doses.

Table 2 shows the whole‐body 3D gamma passing rates for all patient. The average whole‐body 3D gamma passing rates (3%/3 mm) were 99.1 ± 0.6%, 99.7 ± 0.3%, and 100.0 ± 0.1% for 3DVH–HS, 3DVH–NS, and DBDR, respectively. The results of DVH‐based QA for each DVH dose index and the confidence limits are summarized in Table 3. These dose differences varied for each reconstruction method and DVH dose index. The confidence limits in this study were within 9.67% for 3DVH–HS, 9.72% for 3DVH–NS, and 3.64% for DBDR.

**Table 2:** 3D global gamma passing rates (Threshold = 10%) between TPS‐calculated dose and each dose reconstruction method for all patients

Patient No.	Treatment site	Global gamma passing rate (%)								
		1%/2 mm			2%/2 mm			3%/3 mm		
		3DVH‐HS	3DVH‐NS	DBDR	3DVH‐HS	3DVH‐NS	DBDR	3DVH‐HS	3DVH‐NS	DBDR
1	Prostate	96	94.4	99.8	99	98.9	100	99.8	99.8	100
2	Prostate	94	93.8	100	98.2	98.6	100	99.5	99.7	100
3	Prostate	94.2	94.5	100	98.4	99.1	100	99.6	99.9	100
4	Prostate	89.3	91	100	95.5	98.1	100	98.6	99.7	100
5	Prostate	95.2	95.3	100	98.2	99.4	100	99.4	99.9	100
6	Prostate	96.2	96.1	100	98.8	99.4	100	99.6	99.8	100
7	Prostate	96.4	96.5	100	98.9	98.9	100	99.6	99.8	100
8	Prostate	96.3	96.1	100	98.6	99.3	100	99.5	99.8	100
9	Prostate	94.3	94.1	100	98.2	99	100	99.4	99.8	100
10	Prostate	93	94.2	100	97.4	99.2	100	99.1	99.8	100
11	Prostate	90.3	91.1	100	95.1	97.2	100	98	99.3	100
12	Prostate	93.7	93.9	100	97.9	99	100	99.3	99.7	100
13	Prostate	96.1	96.4	100	98.5	99.2	100	99.4	99.8	100
14	Prostate	90.1	90.2	100	96	97.9	100	98.4	99.5	100
15	Prostate	87.9	87.3	100	93.7	95.7	100	97.2	98.7	100
16	Head and neck	89.6	92.8	98.3	95	98	99.8	98.8	99.7	100
17	Head and neck	87.9	91.9	98.8	96.3	98.5	99.9	99.5	99.9	100
18	Head and neck	82	86.8	95.9	92.4	96.2	98.7	99.1	99.7	99.9
19	Head and neck	86.8	93	98	94	98.3	99.7	99	99.9	100
20	Esophagus	89.7	86.8	98.5	97.7	97.4	99.8	99.4	99.2	100
21	Esophagus	88.2	90.9	92	96.1	98.9	97.8	99.4	99.9	99.5
22	Esophagus	73	75.5	99.4	87.1	94.1	100	98	99.6	100
23	Esophagus	88.3	92	98.4	97.7	99.5	99.9	99.8	100	100
Average		90.8	91.9	99.1	96.5	98.3	99.8	99.1	99.7	100.0
S.D.		5.3	4.5	1.8	2.7	1.3	0.5	0.6	0.3	0.1

3DVH‐HSm, High Sensitivity dose morphing in 3DVH software; 3DVH‐NS, Normal Sensitivity dose morphing in 3DVH software; DBDR, in‐house Dynalog‐based dose reconstruction method.

**Table 3:** DVH dose index analysis between TPS‐calculated dose and each reconstructed dose (average ± SD, %), and confidence limits for all DVH parameters calculated by the eq. (1) in the text

Treatment site	Structure	Dose index	Difference to TPS calculated value (%)			Confidence limits (%)		
			3DVH‐HS	3DVH‐NS	DBDR	3DVH‐HS	3DVH‐NS	DBDR
Prostate (*n*=15)	PTV	D_mean_	1.52 ± 0.50	−0.14 ± 0.55	−0.03 ± 0.07	2.49	1.22	0.17
		D_95_	0.91 ± 0.55	−0.82 ± 0.60	−0.07 ± 0.11	1.99	1.99	0.29
		D_max_	6.24 ± 1.70	4.73 ± 1.66	−0.15 ± 0.36	9.57	7.98	0.86
	Rectum wall	D_mean_	1.74 ± 0.59	0.04 ± 0.48	−0.25 ± 0.14	2.91	0.98	0.52
		V_35_	0.31 ± 1.45	−1.91 ± 1.13	−0.39 ± 0.31	3.16	4.12	0.99
		V_55_	4.21 ± 1.54	1.63 ± 1.39	−0.51 ± 0.36	7.23	4.35	1.21
	Bladder wall	D_mean_	1.71 ± 0.67	1.34 ± 1.23	−0.15 ± 0.14	3.02	3.75	0.43
		V_35_	0.44 ± 0.58	−0.55 ± 0.59	−0.15 ± 0.15	1.58	1.71	0.44
		V_55_	2.34 ± 0.90	0.72 ± 0.95	−0.38 ± 0.22	4.09	2.59	0.82
Head and Neck (*n*=4)	PTV	D_50_	1.43 ± 0.25	0.37 ± 0.42	0.15 ± 0.04	1.92	1.19	0.23
		D_max_	3.76 ± 0.92	2.98 ± 0.78	0.76 ± 0.61	5.57	4.51	1.95
	Brain stem	D_max_	3.01 ± 1.07	1.81 ± 0.48	0.17 ± 0.32	5.12	2.75	0.80
	Spinal cord	D_max_	1.57 ± 1.20	0.45 ± 1.16	0.18 ± 0.24	3.92	2.73	0.65
	Right parotid	D_max_	2.76 ± 0.90	1.76 ± 0.78	0.37 ± 0.36	4.52	3.29	1.07
		D_mean_	0.83 ± 0.7	−0.57 ± 0.82	1.49 ± 1.08	2.21	1.03	3.62
	Left parotid	D_max_	2.33 ± 1.16	1.63 ± 1.59	−0.12 ± 0.54	4.60	4.74	0.95
		D_mean_	0.16 ± 0.66	−0.44 ± 0.90	0.05 ± 0.44	1.45	1.32	0.92
Cervical Esophagus (*n*=4)	PTV	D_50_	1.87 ± 0.61	1.25 ± 0.81	0.06 ± 0.11	3.07	2.83	0.27
		D_max_	5.44 ± 2.16	4.53 ± 2.65	0.12 ± 0.45	9.67	9.72	1.01
	Lung	D_mean_	1.57 ± 0.39	1.81 ± 0.65	−0.03 ± 0.11	2.33	3.10	0.18
		V_20_	3.26 ± 1.49	3.49 ± 1.89	−0.05 ± 0.08	6.18	7.20	0.11
	Spinal cord	D_max_	2.18 ± 1.93	0.79 ± 2.53	1.09 ± 1.30	5.97	5.76	3.64

3DVH‐HS, High Sensitivity dose morphing in 3DVH software; 3DVH‐NS, Normal Sensitivity dose morphing in 3DVH software; DBDR, in‐house Dynalog‐based dose reconstruction method.

**Figure 3:**
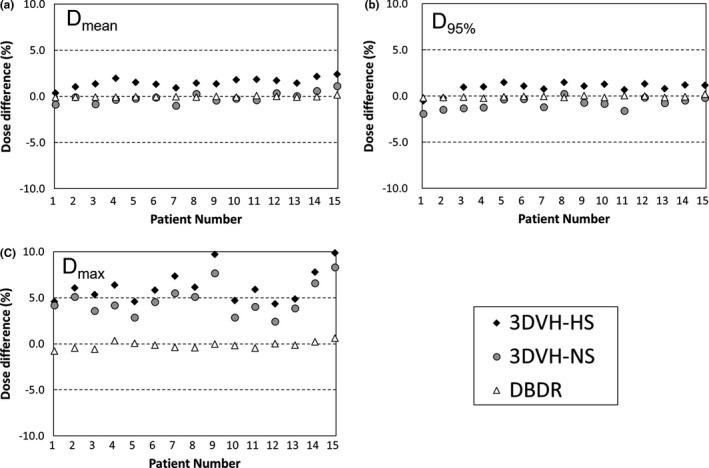
Dose differences in parameter mean dose (D_mean_) and maximum dose (D_max_) for the target volume (PTV) between TPS‐calculated dose and each reconstructed dose, for all prostate patients. The DBDR dose showed good agreement to the reference TPS‐calculated dose in all DVH parameters, while 3DVH doses showed some difference to the reference dose in a specific DVH parameter such as maximum dose.

**Figure 4:**
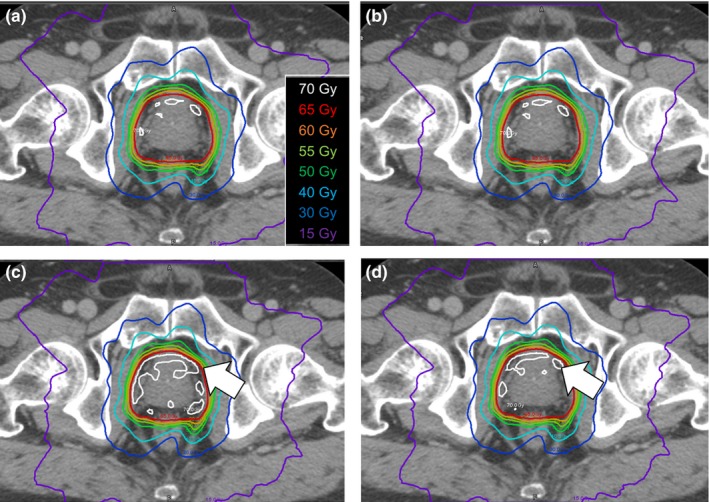
Dose distribution of each reconstruction method for the typical prostate cancer case (patient 8). Reference TPS‐calculated dose (a), reconstructed dose using DBDR (b), 3DVH‐HS (c), and 3DVH‐NS (d) are represented. The DBDR dose was similar to the reference TPS‐calculated dose. On the other hand, the high dose region in the target volume that indicated by the white arrows was observed for the 3DVH reconstructed dose.

Figure 3 shows dose differences in parameter mean dose (Dmean) and maximum dose (Dmax) for the target volume (PTV) between TPS‐calculated dose and each reconstructed dose, for all prostate patients. Although there were good agreements between the TPS‐calculated dose and the DBDR dose for all prostate patients, the systematic errors for the D_max_ were observed in 3DVH methods compared with DBDR. Figure 4 shows the typical prostate patient (patient 8) with a trend toward higher dose in the target volume for the 3DVH reconstructed dose. The average differences between the TPS‐calculated dose and reconstructed dose in the PTV mean dose of the prostate patients were 1.52 ± 0.50%, −0.14 ± 0.55%, and −0.03 ± 0.07% for 3DVH–HS, 3DVH–NS, and DBDR, respectively. On the other hand, the average differences between the TPS‐calculated dose and reconstructed dose in the PTV D_max_ of the prostate patients were 6.24 ± 1.70%, 4.73 ± 1.66%, and −0.15 ± 0.36% for 3DVH–HS, 3DVH–NS, and DBDR, respectively.

**Figure 5:**
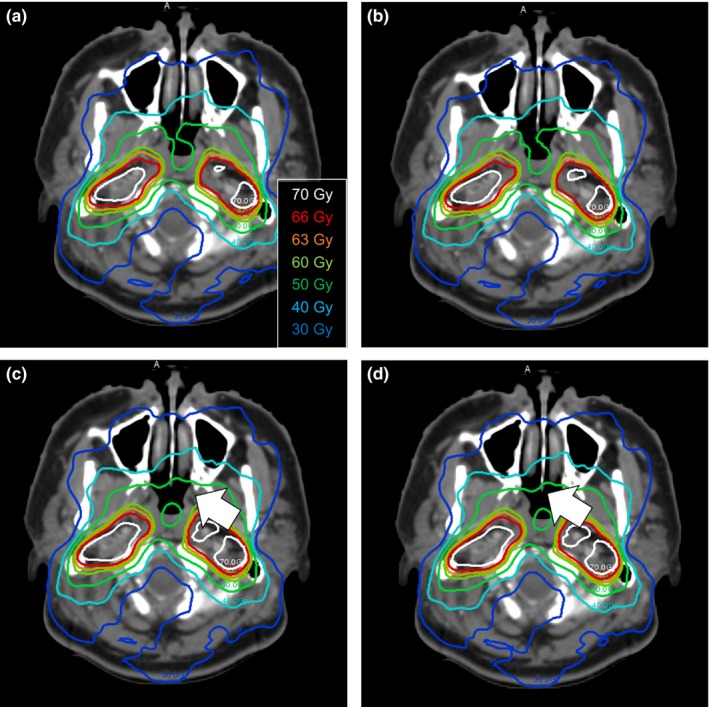
Dose distribution of each reconstruction method for the typical head and neck cancer case (patient 18). Reference TPS‐calculated dose (a), reconstructed dose using DBDR (b), 3DVH‐HS (c), and 3DVH‐NS (d) are represented. The DBDR dose was similar to the reference TPS‐calculated dose. On the other hand, increasing the low dose region in the paranasal sinus that indicated by the white arrows was observed for the 3DVH reconstructed dose.

**Figure 6:**
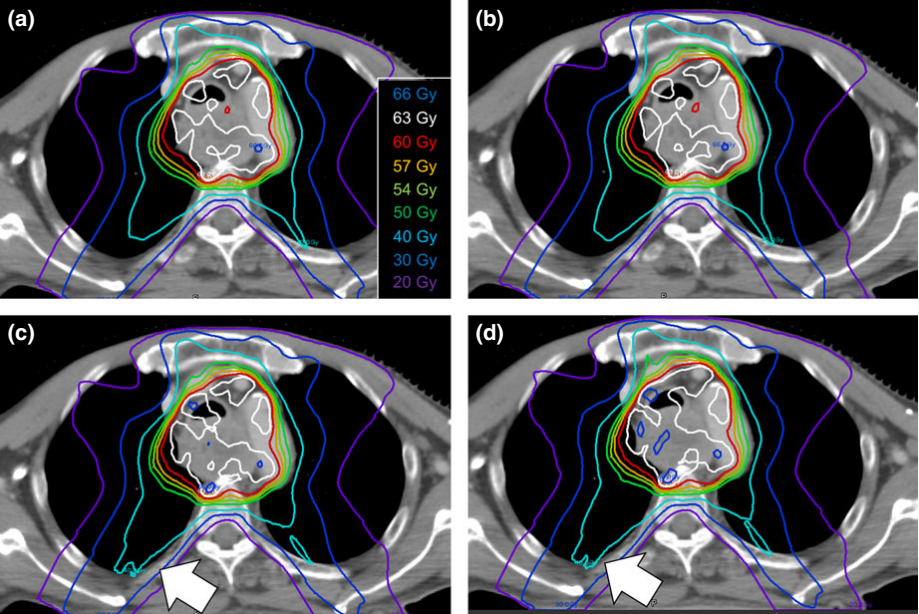
Dose distribution of each reconstruction method for the typical esophagus cancer case (patient 21). Reference TPS‐calculated dose (a), reconstructed dose using DBDR (b), 3DVH‐HS (c), and 3DVH‐NS (d) are represented. The DBDR dose was similar to the reference TPS‐calculated dose. On the other hand, increasing the low dose region in the right lung that indicated by the white arrows was observed for the 3DVH reconstructed dose.

In addition, each reconstructed dose was affected by heterogeneities as shown in Figs. 5 and 6. Especially, increasing low dose region in the organ that includes air cavity such as paranasal sinus, trachea, and lungs was observed. The results of DVH‐based QA for each DVH dose index and the confidence limits for the heterogeneous sites are also summarized in Table 2.

## 4 DISCUSSION

In this study, before comparing two DVH‐based QA methods that were the ArcCHECK‐3DVH system and the in‐house DBDR system, we validated the in‐house DBDR system using MLC error plans, showing that the system was a well‐developed DVH‐based QA tool. Thereafter, we compared these methods using 3D analysis, showing that the ArcCHECK‐3DVH system had some differences from the in‐house DBDR system.

The accuracy of the ArcCHECK‐3DVH system has been validated by several authors, such as Olch et al., who used ion chamber and EDR2 film,^4^ and Watanabe et al., who used BANG3 polymer gel dosimeter.^5^ Furthermore, using error‐induced plans, Kadoya et al. reported that 3DVH–NS was better than 3DVH–HS in terms of dose reconstruction accuracy.^6^ These findings were consistent with our results in the present study. Particularly, compared with 3DVH–HS, the 3DVH–NS had a dose distribution that was in good agreement with the TPS‐calculated dose distribution (Fig. 2). In addition, 3DVH methods use the ACPDP model parameters optimized in other facilities beforehand. Therefore, for DVH dose index analysis, the systematic errors for a specific DVH parameter were observed in 3DVH methods than DBDR (Fig. 3). In addition, we evaluated the method under the heterogeneous treatment sites (Figs. 5 and 6). The 3DVH reconstructed dose is calculated by dose ratio map between ACPDP and TPS based on the homogeneity cylindrical phantom.^12^ Therefore, the effect of heterogeneity is not considered under the dose reconstruction, resulting in a change of the dose distribution especially in the heterogeneous region such as paranasal sinus and lungs.

The dose difference of the in‐house DBDR system from the TPS‐calculated dose was smaller than that of the ArcCHECK‐3DVH system. This result was congruent with the results of previous studies.^11^ The improvement in dose reconstruction accuracy may have been due to identical dose calculation algorithm for both treatment planning and DBDR. That is, the errors caused by the different dose calculation algorithms were zero in log file‐based dose reconstruction method. Therefore, in contrast to the 3DVH method, the dose distribution using DBDR method was good agreement to the TPS‐calculated dose even under the heterogeneous situation. Although machine log file‐based dose reconstruction may be useful for patient‐specific QA, only few hospitals use this method. Further evidence is needed to implement this method into clinical practice.

In this study, the measured dose with the ArcCHECK and the machine log file were acquired only once for each plan, respectively. This is a limitation of the study and the reproducibility of each reconstruction method remained to be evaluated in further study. However, in clinical practice, multiple measurements are not allowed depending on the situation such as in vivo QA using the DBDR method with cone‐beam computed tomography. Furthermore, it is important to simplify the procedures to reduce the burden on clinical staff. Therefore, our results will serve to help medical physicists to understand the reliability of once measurement of each dose reconstructed method.

Some previous studies on large planning data (700 cases of Head and Neck VMAT, and 73 cases of prostate VMAT) were reported for the MatriXX‐COMPASS system, one of the DVH‐based QA methods of using MGDR.^14^, ^15^ On the other hand, there have been no large‐scale studies on ArcCHECK‐3DVH system and machine log‐file based dose reconstruction. In this study, we investigated the accuracy of different dose reconstruction methods on 15 prostate, 4 head and neck, and 4 esophagus VMAT patients. We calculated the confidence limit for each DVH‐based QA metrics (Table 3). In terms of tolerance for DVH‐based patient‐specific QA, Visser et al. suggested that action levels may clearly distinguish the role of the medical physicist and radiation oncologist during the QA procedure.^14^ Our results indicate that these confidence limits may be used by medical physicists.

## 5 CONCLUSION

The two DVH‐based QA methods that we evaluated in this study had different dose reconstruction accuracies. Although with some residual dose reconstruction errors, these two methods can be clinically used as effective tools for DVH‐based QA for VMAT.

## CONFLICT OF INTEREST

There is no conflict of interest with regard to this manuscript.
